# Epidermal inclusion cyst in an axillary lymph node with breast cancer: A case report

**DOI:** 10.3892/mco.2024.2769

**Published:** 2024-08-02

**Authors:** Ari M. Abdullah, Sami Saleem Omar, Abdullah A. Qadir, Abdulwahid M. Salih, Lana R.A. Pshtiwan, Ronak Saeed Ahmed, Rawa M. Ali, Hiwa O. Baba, Rebaz O. Mohammed, Halkawt Omar Ali, Fahmi Hussein Kakamad

**Affiliations:** 1 Department of Scientific Affairs, Smart Health Tower, Sulaymaniyah, Kurdistan 46001, Iraq; 2 Department of Pathology, Sulaymaniyah Teaching Hospital, Sulaymaniyah, Kurdistan 46001, Iraq; 3 Kscien Organization for Scientific Research, Sulaymaniyah, Kurdistan 46001, Iraq; 4 Rizgary Oncology Center, Erbil, Kurdistan 44001, Iraq; 5 College of Medicine, University of Sulaimani, Sulaymaniyah, Kurdistan 46001, Iraq; 6 Shahid Nabaz Dermatology, Teaching Center for Treating Skin Diseases, Sulaymaniyah, Kurdistan 46001, Iraq; 7 Hospital of Treatment of Victims of Chemical Weapons, Halabja, Kurdistan 46018, Iraq

**Keywords:** epidermal cysts, sebaceous cysts, infundibular cysts

## Abstract

Epidermal inclusion cyst (EIC) is a benign lesion rarely discovered within lymph nodes. The present case report introduces an EIC incidentally discovered during an axillary lymph node biopsy in a patient with invasive ductal carcinoma of the breast. A 55-year-old woman presented with a breast mass. Ultrasound revealed a suspicious mass, and a core needle biopsy confirmed the diagnosis of invasive ductal carcinoma. Lumpectomy and sentinel lymph node biopsies were performed. Histopathological examination revealed tumor-free lymph nodes, with one of them harboring a keratinous EIC. EICs typically arise from entrapped epidermal cells. Their presence in lymph nodes is exceptionally rare. While the origin of such inclusions remains unclear, various theories exist, including anomalous embryonic development, implantation, and metaplasia. This report highlights the unique presentation of an EIC within an axillary lymph node. Recognizing this entity is crucial to avoid misdiagnosis of malignancy and unnecessary interventions.

## Introduction

Epidermal inclusion cysts (EICs) are benign nodules often misnamed as sebaceous cysts because they are filled with keratin (protein) rather than sebum (oil). They are non-cancerous nodules that develop due to entrapped epidermal cells (1). These cysts typically appear on the scalp, face, neck, trunk, and back (1-3). They rarely occur within lymph nodes, termed nodal epidermal inclusion cysts (NEICs). These have been reported in the pelvic, abdominal, mediastinal, and axillary regions (4). Epidermal inclusion cysts can occur at any age however they are more common between the third and fourth decades of life and predominantly affect males (1). Cutaneous cysts are classified into true cysts and pseudocysts: True cysts are either lined by stratified or non-stratified squamous epithelium, while pseudocysts lack an epithelial lining (1,2). The presence of an epidermal cyst within an axillary lymph node is an infrequent occurrence.

The present study aimed to report a case of an EIC within an axillary lymph node, incidentally discovered during the histopathological study of a sentinel axillary lymph node biopsy in a patient with invasive ductal carcinoma of the breast.

## Case report

### Patient information

A 55-year-old woman, gravida six and para six, presented with a two-week history of a right breast mass. The patient had no notable medical history apart from a total thyroidectomy performed 6 years ago. Family history was positive for both breast and thyroid cancer. Written informed consent was obtained from the patient for the participation in the present study, for the publication of the present case report and for any accompanying images.

### Clinical findings

On the same day of presentation, examination revealed a small, firm, fixed, and painless lump in the upper outer quadrant of the right breast, while no palpable lymphadenopathy or lymph node abnormalities in the axillary region and no skin surface changes, erythema, discharge, or systematic symptoms were observed.

### Diagnostic approach

Laboratory tests were conducted on the same day and they were within the normal ranges. The following day, a breast ultrasound (U/S) was conducted and revealed a small, irregular, and hypoechoic mass measuring 7x6 mm at 10 o'clock in the posterior depth of the right breast. Axillary sonography revealed no abnormal lymph nodes (BIRADS score U6) and core needle biopsy of the mass confirmed the diagnosis of invasive ductal carcinoma.

### Therapeutic intervention

**Figure 1:**
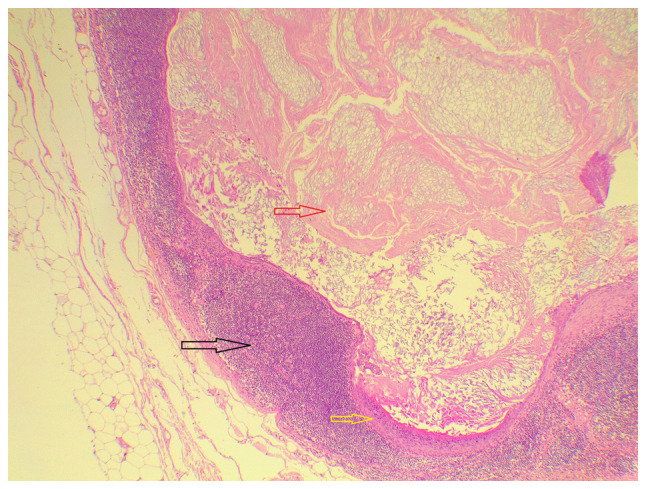
Hematoxylin and eosin staining of an epidermal inclusion cyst. The tissue section reveals a lymph node (dark arrow) containing a cyst lined by stratified squamous epithelial cells with a thick granular layer (yellow arrow) and laminated keratin in the lumen (red arrow) (x40 magnification).

After 1 week of the patient's visit to the hospital, wide local excision of the lump was performed with an axillary lymph node sentinel biopsy. Gross examination revealed a 1.3-cm poorly defined, firm, white, spiculated mass within the breast. The axillary lymph nodes, with two stained and one unstained, appeared entirely normal. The tissue samples underwent fixation in formalin and embedding in paraffin. Sections of 4-µm thickness were then cut using a microtome and stained using conventional hematoxylin and eosin stain from MilliporeSigma. This staining procedure occurred at room temperature over a duration of 65 min, utilizing a Tissue-Tek Prisma Plus Automated slide stainer from Sakura Finetek Europe B.V. Examination of the stained sections was conducted using an Olympus BX-51 microscope equipped with a camera adaptor (Olympus U-TV0.5XC-3; Olympus Corporation) to capture images. Microscopic examination revealed a unifocal invasive ductal carcinoma of no specific type, moderately differentiated, without lymph vascular invasion, associated with low-grade ductal carcinoma *in situ* within the tumor boundary. All three lymph nodes were tumor-free. Notably, one the stained lymph nodes, however, contained a unilocular cyst filled with laminated keratin and lined by stratified squamous epithelium with a prominent granular layer and a surrounding giant cell reaction (Fig. 1). These findings were consistent with an intranodal EIC that had no relation to the invasive ductal carcinoma within the ipsilateral breast.

### Follow-up and outcome

The post-operative period was uneventful, and the patient was scheduled for follow-up.

## Discussion

Epidermal inclusion cysts, referred to by various names such as epidermal cysts, sebaceous cysts, or infundibular cysts, are non-cancerous sacs that develop due to the overgrowth of skin cells within the dermal or subcutaneous layers, resulting in the creation of a cyst filled with keratin (5,6). These cysts can manifest in various locations across the body, typically appearing as nodules directly beneath the skin surface, and often exhibiting a visible central punctum. Epidermal cysts primarily contain keratin rather than sebum within their centers. The cysts do not originate from sebaceous glands; hence, they are distinct from sebaceous cysts. While the terms ‘sebaceous’ and ‘epidermoid’ cysts should not be used interchangeably, in clinical settings, this distinction is often overlooked (7).

The formation of an EIC typically involves a combination of factors such as trauma, epithelial proliferation, or minimal inflammation. Additionally, since EICs are often asymptomatic and slow-growing, patients may not readily associate the lesion with any previous trauma they may have experienced (8). In the present case report, the patient did not report any history of trauma or surgical procedure in the region of the cyst.

Benign inclusions within lymph nodes were initially documented by Ries in 1897, who categorized them into three groups: Epithelial, nevomelanocytic, and decidual (9). Fellegara *et al* (10) further classified nodal epithelial inclusions into glandular, squamous, and mixed types. The lymph nodes most commonly affected vary depending on the type of heterotopic inclusion. For instance, axillary nodes often contain breast tissue and nevus cells, while cervical nodes may harbor salivary glands or thyroid tissue. Pelvic and retroperitoneal nodes may exhibit decidual tissue, intestinal glands, or mesothelial cells. The origin of these epithelial inclusions remains a subject of debate, with proposed theories including embryogenic, implantation (iatrogenic), and metaplastic origins (11).

Complications associated with EICs include rupture, inflammatory alterations, or the formation of abscesses, which can pose challenges in achieving an accurate imaging diagnosis. In rare instances (~2% of cases), malignant transformation into squamous cell carcinoma within the EIC wall has been reported. Suspicion of malignant degeneration should arise if solid nodules are detected alongside the cyst wall (5). In the present study, clinical signs of infection or malignant change were absent.

Ultrasonography is prioritized as the primary imaging method for assessing superficial soft tissue masses, including those within the skin, due to its high-resolution capabilities, absence of radiation, cost-effectiveness, and widespread availability. This makes it preferable over other modalities such as computed tomography or magnetic resonance imaging. When assessing palpable lesions in the axilla, ultrasound serves to identify and pinpoint the lesion, distinguishing between lymph nodes and soft tissue masses that might require preoperative tissue confirmation. In cases reported in reputable scientific literature from non-predatory journals where ultrasound reveals characteristic or distinctive features, a precise sonographic diagnosis can be achieved without the need of a biopsy, potentially averting further unnecessary examinations (5,12). During ultrasonography, an EIC might exhibit a solid and well-defined structure, appearing complex or heterogeneous. In certain instances, it may display a distinctive concentric pattern resembling onion rings, with alternating hypoechoic and hyperechoic layers (13). In a study by Abu-Mandeel *et al* (3), which involved a 41-year-old patient, the ultrasound revealed a hypo-echoic subcutaneous mass in the right axilla with internal foci corresponding to calcifications consistent with the present study, in which U/S of the breast and axilla revealed a small, irregular, hypoechoic mass measuring 7x6 mm within the breast with no abnormalities in the axillary lymph nodes.

Due to its wide prevalence, notable advancements have been made in diagnosing and treating breast cancer across sexes. Despite this, several critical clinical and scientific challenges persist regarding prevention, diagnosis, prognosis, recurrence, and treatment options. These challenges are further highlighted by the occasional coexistence of benign EIC with breast malignancies (2,14). Typically, small, uncomplicated cysts do not require treatment. However, if removal is desired, it can be achieved through a straightforward surgical excision, ensuring the complete removal of the cyst along with its intact wall (10). Benign epithelial inclusion cysts can occur in patients with or without associated breast pathology and, rarely, they are discovered within lymph nodes alongside metastatic breast carcinoma. Fisher *et al* (15) documented a case involving a 55-year-old woman diagnosed with infiltrating ductal carcinoma. During axillary dissection, two lymph nodes were extracted, confirming metastatic carcinoma and associated epithelial inclusion cysts. In a study reported in the literature, a histopathological examination of a sample from a 41-year-old patient, stained with hematoxylin and eosin, revealed depiction of the cyst wall, presence of squamous epithelial lining, and accumulation of laminated keratin layers (3). Additionally, in a study which reported multiple bilateral EICs, a biopsy revealed a cyst with a lining of multiple layers of squamous epithelial cells and a cavity filled with keratin (16). In line with these studies, in the current case, EIC was diagnosed incidentally in the sentinel lymph node biopsy of a case with invasive ductal carcinoma of the breast; three lymph nodes were isolated, two of which were stained (with the methylene blue dye used for the sentinel biopsy procedure), and all were free from tumors. Notably, one of the stained lymph nodes, however, revealed a keratinous EIC with a giant cell reaction.

The coexistence of benign EICs with breast malignancies presents diagnostic challenges for pathologists, radiologists and clinicians. Pathologists should be vigilant in histopathological examination to identify incidental EICs within lymph nodes, necessitating thorough communication with clinicians. Radiologists play a pivotal role in recognizing the sonographic features of EICs during ultrasound evaluation, aiding in accurate diagnosis and treatment planning. Clinicians should consider the potential presence of concurrent benign lesions such as EICs when interpreting diagnostic findings, emphasizing multidisciplinary collaboration to ensure comprehensive patient care and optimal outcomes. Integration of these recommendations into clinical practice can enhance diagnostic accuracy and improve patient management in breast cancer cases. A limitation of the present study is the absence of ultrasound imaging. Additionally, although a small, firm, fixed, and painless lump in the upper outer quadrant of the right breast was revealed upon examination, representative images of this mass are unavailable. Regrettably, due to the lack of captured ultrasonographic images at the time of examination and the early stages of electronic medical record adoption in the authors' region, technical challenges are still faced, resulting in some images being unretrievable.

In conclusion, it was deemed valuable to document this rare instance of an EIC found within an axillary lymph node to raise awareness among pathologists regarding this unusual benign condition within lymph nodes. Recognizing this rare entity can help prevent misdiagnosis of a malignant lesion, particularly metastatic carcinoma in this context, thus avoiding unnecessary treatments.

## Data Availability

The data generated in the present study may be requested from the corresponding author.
